# Enhancing Water Purification by Integrating Titanium Dioxide Nanotubes into Polyethersulfone Membranes for Improved Hydrophilicity and Anti-Fouling Performance

**DOI:** 10.3390/membranes14050116

**Published:** 2024-05-17

**Authors:** Ayesha Bilal, Muhammad Yasin, Faheem Hassan Akhtar, Mazhar Amjad Gilani, Hamad Almohamadi, Mohammad Younas, Azeem Mushtaq, Muhammad Aslam, Mehdi Hassan, Rab Nawaz, Aqsha Aqsha, Jaka Sunarso, Muhammad Roil Bilad, Asim Laeeq Khan

**Affiliations:** 1 Department of Chemistry, COMSATS University Islamabad, Lahore Campus, Lahore 54000, Pakistan; ayeshabilal288@gmail.com (A.B.); mazhargilani@cuilahore.edu.pk (M.A.G.); 2 Department of Chemical Engineering, COMSATS University Islamabad, Lahore Campus, Lahore 54000, Pakistan; azeemmushtaq@cuilahore.edu.pk (A.M.); maslam@cuilahore.edu.pk (M.A.); 3 Department of Chemistry and Chemical Engineering, Lahore University of Management Sciences (LUMS), Lahore 54792, Pakistan; faheem.akhtar@lums.edu.pk; 4 Department of Chemical Engineering, Faculty of Engineering, Islamic University of Madinah, Madinah 42351, Saudi Arabia; ham7171@gmail.com; 5 Department of Chemical Engineering, Faculty of Mechanical, Chemical and Industrial Engineering, University of Engineering and Technology, Peshawar 25120, Pakistan; m.younas@uetpeshawar.edu.pk; 6 School of Energy and Environment, City University of Hong Kong, Kowloon Tong, Hong Kong SAR, China; 7 Department of Chemistry, University of Baltistan, Skardu 16100, Pakistan; mehdi.hassan@uobs.edu.pk; 8 Center for Applied Mathematics and Bioinformatics (CAMB), Gulf University for Science and Technology, Hawally 32093, Kuwait; nawaz.r@gust.edu.kw; 9 Department of Bioenergy Engineering and Chemurgy, Faculty of Industrial Technology, Institute Teknologi Bandung, Bandung 40132, Indonesia; aqsha@itb.ac.id; 10 Research Centre for Sustainable Technologies, Faculty of Engineering, Computing and Science, Swinburne University of Technology, Jalan Simpang Tiga, Kuching 93350, Sarawak, Malaysia; jsunarso@swinburne.edu.my; 11 Faculty of Integrated Technologies, Universiti Brunei Darussalam, Gadong BE 1410, Brunei; roil.bilad@ubd.edu.bn

**Keywords:** mixed matrix membranes, TiO_2_ nanotubes, water purification

## Abstract

Water pollution remains a critical concern, one necessitated by rapidly increasing industrialization and urbanization. Among the various strategies for water purification, membrane technology stands out, with polyethersulfone (PES) often being the material of choice due to its robust mechanical properties, thermal stability, and chemical resistance. However, PES-based membranes tend to exhibit low hydrophilicity, leading to reduced flux and poor anti-fouling performance. This study addresses these limitations by incorporating titanium dioxide nanotubes (TiO_2_NTs) into PES nanofiltration membranes to enhance their hydrophilic properties. The TiO_2_NTs, characterized through FTIR, XRD, BET, and SEM, were embedded in PES at varying concentrations using a non-solvent induced phase inversion (NIPS) method. The fabricated mixed matrix membranes (MMMs) were subjected to testing for water permeability and solute rejection capabilities. Remarkably, membranes with a 1 wt% TiO_2_NT loading displayed a significant increase in pure water flux, from 36 to 72 L m^2^ h^−1^ bar^−1^, a 300-fold increase in selectivity compared to the pristine sample, and a dye rejection of 99%. Furthermore, long-term stability tests showed only a slight reduction in permeate flux over a time of 36 h, while dye removal efficiency was maintained, thus confirming the membrane’s stability. Anti-fouling tests revealed a 93% flux recovery ratio, indicating excellent resistance to fouling. These results suggest that the inclusion of TiO_2_ NTs offers a promising avenue for the development of efficient and stable anti-fouling PES-based membranes for water purification.

## 1. Introduction

Access to clean drinking water has become a global concern with the abrupt increase in population and energy consumption due to rapid urbanization and industrialization [1,2]. This surge has led to an exponential increase in wastewater generation, which not only contributes to environmental degradation but also poses serious health risks through water pollution [3,4]. Traditional water treatment methods such as coagulation, flocculation, adsorption, and ion exchange have been proven inadequate for effective removal of a wide range of pollutants, including salts, heavy metals, and hazardous dyes [5]. Consequently, there is an emergent need for more sustainable, efficient, and cost-effective water purification technologies. Membrane-based processes have emerged as a viable solution, offering advantages in treatment efficiency, operational cost, and energy consumption [6,7]. Among the different membrane processes—ultrafiltration (UF), nanofiltration (NF), and reverse osmosis (RO)—NF stands out as particularly promising. This is due to its optimal pore size range (1–2 nm), which enables effective salt removal under moderate operating pressures (5–20 bar) [8,9,10].

While polymeric membranes dominate the landscape of NF, these materials have their own limitations. Commonly used polymers in membrane fabrication include polyether sulfone (PES), polyimide (PI), polysulfone (PS), polyvinyl chloride (PVC), etc. [11,12]. Among these, PES stands out for its excellent chemical resistance, thermal stability, oxidation resistance, and mechanical properties. However, PES membranes are often hampered by their intrinsic hydrophobicity, which is attributed to the alternating ether and sulfone linkages between aromatic rings. This hydrophobic nature leads to membrane fouling, thereby compromising both separation and purification efficiencies [13]. To overcome these limitations, mixed matrix membranes (MMMs) have gained prominence as a promising alternative [14]. These membranes synergistically combine an organic polymeric matrix with inorganic nanofillers, offering enhanced performance attributes [15]. Various methods for fabricating MMMs include blending [16], coating [17], graft polymerization [18], and interfacial polymerization (IP) [19]. Of these, blending has emerged as the most practical approach, owing to its cost-effectiveness, ease of control, and environmental compatibility. This method can be further categorized into two strategies: blending nanomaterials within the MMM through incorporation in the polymer casting solution and assembling nanomaterials atop the membrane’s surface [20]. The compatibility between the polymeric matrix and the nanomaterials often poses a challenge, leading to undesirable defects and macrovoids in the membrane structure [21]. The variety of viable inorganic nanomaterials embedded in MMMs has expanded considerably, and now includes not only nanoparticles but also sulfides, nitrides, and oxides [22]. Various nanomaterials, such as carbon nanotubes (CNTs) [23], graphene oxide (GO) nanoparticles [24], and titanium dioxide (TiO_2_) nanoparticles and nanotubes (NTs) [25] have been employed to enhance the structural and functional properties of MMMs. 

TiO_2_ has gained considerable attention as a high-performance material, owing to its unique attributes such as high photo-reactivity, superhydrophilicity, non-toxicity, long-term stability, and cost-effectiveness [26]. Recent advancements in membrane technology have resulted from this increased focus on TiO_2_-based MMMs with the goal to enhance hydrophilicity and reduced fouling [22,27,28,29]. For instance, Farahani et al. (2018) developed polyvinylidene fluoride (PVDF)-based MMMs integrated with cloisite 30B clay, carboxyl-functionalized multi-walled carbon nanotubes (MWCNTs-COOH), SiO_2_, and TiO_2_. Their work demonstrated a remarkable 135% improvement in pure water permeance (PWP) accompanied by a nearly 100% Bovine Serum Albumin (BSA) rejection, and a 33% flux recovery ratio [30]. In another study, Jhaveri et al. (2017) fabricated GO-TiO_2_ nanosheets blended with PVC membranes, achieving a pure water flux (PWF) of nearly 105 LMH, 99.8% humic acid rejection, and a 90% flux recovery ratio [31]. Vatanpour et al. (2012) evaluated three different types of TiO_2_ nanoparticles (P25, PC105, and PC500) of varying sizes in PES-based NF membranes. Using whey protein as the foulant, their membranes exhibited a PWF of 7.2 LMH and a 90.8% flux recovery ratio [32].

Despite the prevalent use of TiO_2_ in MMMs, owing to the hydrophilic nature of the TiO_2_, these membranes face the typical trade-off between selectivity and permeability given their tendency to form rapid aggregates, leading to poor polymer–filler interfaces [33,34]. Due to their unique cylindrical structure, the exploitation of TiO_2_ in the morphological shapes of nanotubes (TiO_2_NTs) is expected to offer enhanced selectivity and permeability. TiO_2_NTs offer distinct advantages owing to their high aspect ratio and superior mechanical strength. Moreover, their unique open-ended nanotubular structure—with inner and outer diameters typically ranging from 3 to 5 and 10 to 13 nm, respectively—makes them a promising candidate for a more robust membrane with enhanced morphological characteristics [35,36]. For example, the incorporation of TiO_2_NTs into polymeric membranes has the potential to usher in a new generation of membranes characterized by enhanced selectivity and permeability, reduced fouling, and robust mechanical properties. Their inherent attributes, such as high porosity, thermal stability, and mechanical strength, as well as their large surface area, make TiO_2_NTs a crucial component for advancing membrane performance. Shaban et al. doped PES membranes with various loadings of TiO_2_NTs and evaluated the performance of these membranes in RO applications [37]. When using PES infused with 0.8% TiO_2_NTs, the membranes demonstrated a notable salt rejection of 98% and a permeate flux of 5.45 Kg m^−2^ h^−1^ in brackish water studies. Mahdi et al. employed a non-solvent induced phase separation (NIPS) approach to modify PES ultrafiltration membranes with different concentrations of TiO_2_NTs. Zeta potential and water contact angle (WCA) measurements showed enhanced hydrophilicity and surface negative charge in TiO_2_NTs/PES nanocomposite membranes compared to unmodified PES membranes. The membranes showed higher organic matter rejection and PWF than those of the nascent PES membrane. The addition of TiO_2_NTs at 1 wt% provided a maximum PWF (82 L/m^2^ h at 40 psi), organic matter rejection (53.9%), and anti-fouling properties (29% improvement in comparison to pristine PES membrane) [38].

To the best of our knowledge, this study is the first comprehensive investigation into the incorporation of TiO_2_NTs into PES NF membranes specifically designed for advanced water purification applications. While previous research has explored the general use of TiO_2_ in membrane technology, no studies have delved into the unique synergistic benefits that TiO_2_NTs could offer when embedded in a polymer matrix for NF applications. Our work seeks to fill this critical gap in the literature. The central aim of this research is to achieve a step-change improvement in membrane performance metrics—specifically, permeability, selectivity, and anti-fouling properties—while ensuring long-term operational stability. We hypothesize that the nanotubular morphology and unique physicochemical attributes of TiO_2_NTs can reinforce the PES membranes at the molecular level, thereby enhancing their water treatment efficacy. Our multi-dimensional approach involves exhaustive characterization of the synthesized TiO_2_NTs, followed by their incorporation into PES-based MMMs through a phase inversion technique. We then carried out a rigorous set of performance evaluations covering PWF, ion (NaCl and MgSO_4_) rejection, molecular weight cut-off (MWCO) determination, and anti-fouling properties. This study thus stands to make a pivotal contribution to water treatment technologies, setting new benchmarks for membrane efficiency and durability. Through an elaborative exploration of these novel membranes, this study aims to provide both a scientific and a practical foundation for their potential large-scale application in addressing burgeoning water-purification needs. 

## 2. Materials and Methods

### 2.1. Materials

PES polymer (MW 58,000 g/mol) was purchased from BASF Co., Germany. TiO_2_ nanoparticles (TiO_2_NPs) (Anatase with MW 79.88 g/mol and 98.0% pure), Methylene Blue (MW 373.9 g/mol), and Congo Red (MW 696.68 g/mol) were purchased from Daejung, Gyeonggi, Republic of Korea. 1.4-Dioxane and aluminum chloride were purchased from CARLO ERBA Reagents (Le Vaudreuil, France). N, N-dimethylformamide (DMF), Bovine Serum Albumin (BSA), magnesium sulfate (MgSO_4_), sodium chloride (NaCl), methanol, ethanol, propanol, and hexane (analytical reagent grade) were bought from SIGMA ALDRICH, Hamburg, Germany. Hydrochloric acid (HCL), sodium hydroxide (NaOH), sodium arsenate and chromium chloride were purchased from SIGMA ALDRICH, USA. Rose Bengal (MW 1017.65 g/mol) was obtained from Applichem Panreac, Germany. Deionized (DI) water produced in the laboratory was used during experimentation and analysis. 

### 2.2. Preparation of TiO_2_NTs

TiO_2_NTs were prepared by an alkaline hydrothermal treatment of TiO_2_ NPs based on the protocol earlier reported by Akhtar, F. H., et al. [39]. Initially, 2 g of TiO_2_ NPs were dispersed in 85 mL of a 10 M NaOH solution. This mixture was then mechanically stirred for 24 h at room temperature (~22 °C), forming a homogeneous, white-colored suspension of TiO_2_ particles. Subsequently, the well-dispersed suspension was transferred to a Teflon-lined autoclave and subjected to hydrothermal treatment at 160 °C for 72 h. After the completion of the hydrothermal process, the autoclave was allowed to cool to room temperature, yielding a white, gel-like substance. This gel was immediately transferred to a beaker containing 250 mL of a 0.1 M HCl solution and stirred at room temperature for an additional 24 h to adjust the pH to a neutral level, compensating for the alkaline conditions introduced by residual NaOH. Finally, the purified TiO_2_NTs were dried at 60 °C for 24 h, followed by a secondary drying phase at 100 °C for 6 h to ensure the complete removal of residual moisture. The resultant TiO_2_NTs were then characterized as to their physicochemical properties. A schematic representation of the stepwise synthesis process is provided in Figure S1.

### 2.3. Fabrication of PES Membranes and TiO_2_ NPs/NTs Based MMMs

To evaluate the effectiveness of NT-based fillers in comparison to NPs, MMMs were fabricated using both TiO_2_ NPs and NTs. Different filler concentrations, including 0, 0.25, 0.5, 1, and 1.5%, were used to evaluate their impacts on membrane performance. Flat-sheet asymmetric PES membranes, along with their TiO_2_ NPs- and NTs-infused counterparts, were synthesized employing the NIPS technique. A schematic representation of this process is presented in Figure S2. The polymer casting solutions were formulated by solubilizing 20 wt% PES in a solvent mixture of 1,4-dioxane and dimethylformamide (DMF) at a mass ratio of 1:3. This solution was stirred continuously at room temperature for 36 h to ensure homogeneity and then degassed for an additional 24 h to remove any trapped air bubbles. Subsequently, the polymer dope was spread on a glass plate pre-covered with a non-woven polypropylene fabric (Novatex-2471, Freudenberg Filtration Technologies, Weinheim, Germany). An Elcometer Film Applicator was used to ensure a uniform thickness during casting. Following a 30 s interval for solvent evaporation, the wet films were then transferred into a water coagulation bath for a minimum of 10 min to initiate phase inversion. The resultant membranes were preserved in DI water until further testing.

For the fabrication of TiO_2_ NPs- and NTs-modified PES MMMs, varying filler loadings (0.25, 0.5, 1, and 1.5 wt%) were first dispersed in the solvent mixture under continuous stirring for one h. A stepwise addition of 20 wt% PES was then carried out: initially, 10% of the polymer was added, and the mixture was stirred for 2 h; this was followed by the addition of an additional 20%, followed by a similar stirring period. The remaining polymer was added last and the mixture was then stirred for 24 h to achieve a uniform mixture. The prepared casting solutions were then used to fabricate MMMs following the NIPS process, as previously described. The compositions of all membranes are summarized in Table 1.

### 2.4. Characterization

To evaluate the physicochemical properties of the synthesized filler particles and MMMs, a comprehensive suite of analytical techniques was employed.

Chemical composition and functional group identification were executed using a Thermo-Nicolet 6700 P FTIR spectrometer. The FTIR spectra were recorded over a wavenumber range of 4000–500 cm^−1^, with a resolution of 0–100 nm and an averaging of 25 scans. Crystallography and phase purity of the TiO_2_NPs, TiO_2_NTs, and MMMs were ascertained via XRD analysis using a Philips PAN-analytical X’Pert Pro PW3050/60 diffractometer. Operating parameters were set at 45 kV and 40 mA with CuKα radiation (λ = 0.1540 nm). The samples were scanned over a 2θ range of 5–60°, with a step size of 0.03° and a dwell time of 0.65 s per step, total counting time then being 1200 s. Morphological details and filler distributions within the MMMs were characterized by SEM utilizing a Field Emission Scanning Electron Microscope (FEI Nova 450 NanoSEM). For detailed cross-sectional analysis, membrane samples were cryogenically fractured using liquid nitrogen. Pore characteristics of the TiO_2_ NPs and TiO_2_ NTs were investigated using BET analysis. Thermal stability levels of the membranes were quantified using a Thermo Gravimetric Analyzer (TGA) from the SDT Q600 series, employing a temperature ramp from 25 to 900 °C under a nitrogen gas atmosphere. Surface hydrophilicity of the fabricated membranes was assessed by a Biolin Scientific Attention Theta Flex Analyzer contact angle meter, employing the sessile drop method. Measurements were conducted by applying a 4 μL droplet onto the membrane samples using a micro-syringe. To mitigate variability, five distinct measurements were taken for each membrane type and the average values have been reported.

For the evaluation of average porosity of a membrane (ε), the following equation was used, as previously reported in [40]:

(1) $$\varepsilon =\;\frac{\omega_{1}-\omega_{2}}{Ald_{w}}$$

In Equation (1), the variables ω_1_ and ω_2_ represent the weights of the membrane before and after immersion in water, respectively. These weights are crucial for calculating the average porosity of the membrane. Additionally, *A* is the membrane effective area (m^2^), *d_w_* is the density of water, and *l* is the membrane thickness (m). 

The Guerout–Elford–Ferry equation was used to determine mean pore radius (*r_m_*).

(2) $$r_{m}\;=\;\sqrt{\frac{\left(2.9-1.75\epsilon \right)8\eta lQ}{\in A\Delta P}}$$

where *η* is the viscosity of water (8.9 × 10 ^− 4^ Pa s), Q is the volume of permeated pure water per unit time (m^3^/s), and Δ*p* is the operational pressure (MPa). Three readings were taken to avoid any measurement error, and their average was reported.

This thorough characterization approach ensured the reliability of the synthesized materials and lends validity to the subsequent analysis of their performance in water purification applications.

### 2.5. Membrane Performance Evaluation and Fouling Assessment

To rigorously evaluate the performance attributes of membranes, a dead-end filtration cell (Sterlitech Corporation, HP4750 Stirred Cell, USA) was employed. The filtration assembly, with a processing volume of 300 mL and an effective membrane area of 14.6 cm^2^, was used for pure water permeation. The entire assembly was pressurized using a nitrogen cylinder, maintaining an operational pressure of 8 bar at ambient conditions (27 ± 2 °C). Prior to the performance tests, each membrane underwent a compaction phase at 8 bar for 30 min to attain a stable flux state. Permeance (P) was computed using the following equation:

(3) $$J=\frac{V}{tA\Delta P}$$

where *V* denotes the volume of permeate (L), *A* is effective membrane area (m^2^), *t* is permeation time (h), and Δ*p* is operating pressure (bar).

A feed solution containing 1 g/L each of NaCl and MgSO_4_ was subjected to membrane filtration under the pre-established 8-bar pressure. Solution salinity was quantified using a conductivity meter (HANNA Instruments). The dye concentrations were determined using a UV-vis spectrophotometer (Biotechnology Medical Services UV-1602, USA). The rejection (R) of salts and dyes was obtained by using the following formulas:

(4) $$R=\left(1-\frac{C_{p}}{C_{f}}\right)\times 100$$

where *C_f_* and *C_p_* are the concentrations of salts and dyes in feed and permeate, respectively.

To evaluate the long-term performance consistency, a 36 h filtration test was carried out using a synthesized MMM that exhibited optimal dye removal efficiency. Metrics for dye permeate and removal were collected bi-hourly.

Anti-fouling capabilities of the membranes were evaluated using a 1 g/L BSA (Bovine Serum Albumin) solution. Prior to introducing the BSA, a stable water flux (*J*_1_) was established through pure water filtration for 120 min. The membrane was then exposed to the BSA solution for an additional 90 min, and the BSA flux (*J_p_*) was recorded. Following a thorough rinse with DI water for 30 min, a subsequent water flux (*J*_2_) was measured. 

To quantitatively assess the anti-fouling efficacy, the Flux Recovery Ratio (*FRR*) was computed, as described in Equation (5).

(5) $$FRR=\left(\;\frac{J_{2}}{J_{1}}\right)\times 100$$

Furthermore, fouling attributes were dissected into total fouling ratio (*R_t_*), reversible fouling ratio (*R_r_*), and irreversible fouling ratio (*R_ir_*) using the below equations:

(6) $$R_{t}=\left(1-\frac{J_{p}}{J_{1}}\right)\;\times 100$$

(7) $$R_{r}=\left(\frac{J_{2}-J_{p}}{J_{1}}\right)\times 100$$

(8) $$R_{ir}=\left(\frac{J_{1}-J_{2}}{J_{1}}\right)\times 100$$

This comprehensive testing regime provided an exhaustive evaluation of the MMMs, considering aspects like salinity, dye, long-term performance stability, and anti-fouling characteristics.

## 3. Results and Discussion

### 3.1. Characterization of TiO_2_NPs and TiO_2_NTs

**Figure 1:**
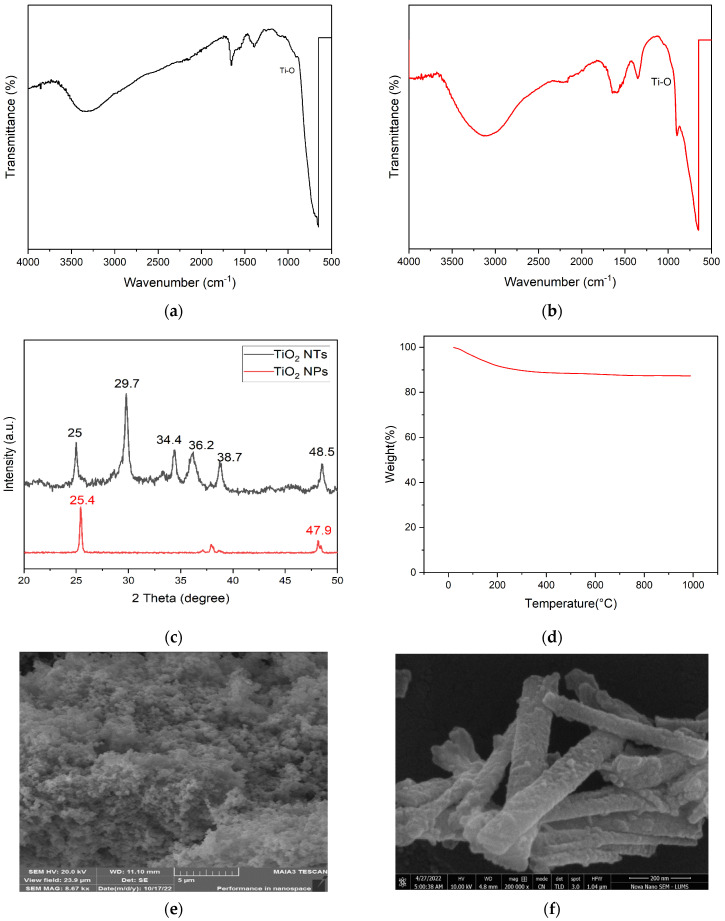

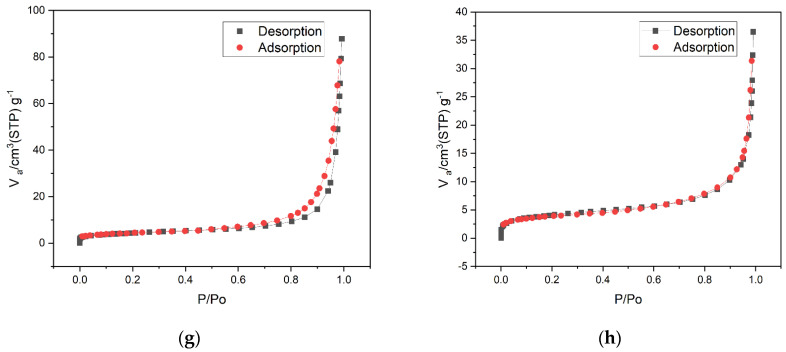
FTIR spectra of (**a**) TiO_2_ nanoparticles and (**b**) nanotubes; (**c**) XRD patterns of TiO_2_NPs and TiO_2_NTs; (**d**) thermogravimetric analysis of TiO_2_NTs; (**e**) SEM image of TiO_2_NPs; (**f**) SEM image of TiO_2_NTs; (**g**) N_2_ adsorption/desorption of TiO_2_NPs; and (**h**) N_2_ adsorption/desorption of TiO_2_NTs.

FTIR analysis was extensively employed to discern the molecular intricacies and the presence of specific functional groups within both the TiO_2_NPs and TiO_2_NTs. In our study, the FTIR spectra delineate distinct features indicative of the inherent chemical compositions and the potential interactions within the synthesized materials. For the TiO_2_ nanoparticles, the FTIR spectrum, as illustrated in Figure 1a, unveils a prominent absorption band spanning the 500–900 cm^−1^ range. This band is principally attributed to the vibrational modes of the Ti-O bonds, which are fundamental to the TiO_2_ chemical structure. The sharpness and intensity of this peak provide evidence of the TiO_2_ nanoparticles. Additionally, the absence of significant peaks outside this range confirms the lack of organic or extraneous inorganic impurities, underscoring the effectiveness of our synthesis and purification protocols.

Transitioning to the TiO_2_ nanotubes, the FTIR spectrum, represented in Figure 1b, presents a marked deviation from that of the nanoparticles. Notably, there is an intensification of the broad band between 3400–3000 cm^−1^, primarily ascribed to the stretching vibrations of hydroxyl (OH) groups. The enhanced presence of these OH groups on the TiO_2_NTs’ surface is indicative of considerable surface modification post-synthesis. This surface hydroxylation is likely a result of the strong hydrothermal conditions employed during the nanotubes’ fabrication, particularly the use of NaOH solutions. The OH functionalities are pivotal, as they significantly augment the surface chemistry of the TiO_2_NTs, enhancing their hydrophilicity and potential interaction capabilities as to polymers and biomolecules. This is critical for their integration into the PES matrix, as these interactions are fundamental in influencing the mechanical properties and stability of the resulting mixed matrix membranes. The observed horizontal peaks and baseline features are significant, as they provide insights into the surface chemistry of the nanotubes. These features often represent the background absorption of the material and are typically present in samples where light-scattering effects are pronounced due to the physical characteristics of the sample, such as particle size and surface irregularities. Moreover, subtle shifts in the peak positions and changes in their intensities within the TiO_2_NT spectrum may hint at structural nuances and variations in the electronic environment surrounding the titanium and oxygen atoms. The observed shifts in the FTIR spectra could be attributed to the nanotubular morphology and the altered crystal field effects, compared to the nanoparticles. A deeper understanding of these variations is important as it helps explain how the structural and electronic properties of TiO_2_NTs enhance their interaction with the polymer matrix and influence the overall performance of the mixed matrix membranes, particularly in terms of hydrophilicity, permeability, and anti-fouling characteristics.

To gain insights into the crystalline features of TiO_2_NPs and TiO_2_NTs, the XRD experiments were carried out, and are presented in Figure 1c. The TiO_2_NTs and NPs both displayed diffraction peaks at angle 2θ = 25° which corresponds with the tetragonal geometry (JCPDS-21-1272) of the anatase phase. Contrastingly, other peaks at 2θ values of 25.4° and 47.9° for TiO_2_ NPs were observed, which clearly indicated amorphous behavior, and various characteristic peaks at 2θ values of 25°, 29.7°, 34.4°, 36.2°, 38.7°, and 48.5° were observed in TiO_2_ NTs.

The TGA results, graphically presented in Figure 1d, offer critical insights into the thermal stability and associated weight-loss behavior of the nanotubes over an extended temperature range. The TGA curve shows that the TiO_2_NTs remain thermally stable up to a remarkable 1000 °C. The TGA results demonstrate the high thermal stability of the TiO_2_NTs, which remains crucial for their applications in membrane technologies. Thermal stability is an important attribute for the operational durability of nanofiltration membranes, especially under conditions that may expose the membranes to elevated temperatures. Our data suggest that the incorporation of TiO_2_NTs could enhance the thermal resilience of the MMMs, thereby potentially extending their operational life and performance stability in various environmental conditions. An initial weight loss was observed which can be attributed to the desorption of water molecules and residual solvents, which were likely entrapped in the NT framework during the synthesis process. The minor initial weight loss, ascribed to water and solvent desorption, does not compromise the material’s structural integrity. 

The SEM analysis provides a profound insight into the morphological distinctions between the TiO_2_ nanoparticles and nanotubes, which are crucial for their application efficacy in mixed matrix membranes. The SEM micrographs, as showcased in Figure 1e,f, present the physical forms and the dimensional attributes of the synthesized materials. Figure 1e exhibits the TiO_2_NPs, which predominantly display spherical and semi-spherical shapes with an apparent size uniformity and a smooth surface topology. This morphology is typical of TiO_2_ nanoparticles synthesized under controlled conditions, and it plays a vital role in their dispersion stability and surface area accessibility. However, the tendency of these nanoparticles to agglomerate, as noted in certain regions of the micrograph, could impede their effective utilization in membrane technologies by affecting the membrane’s porosity and surface properties.

In the SEM image shown in Figure 1f, the TiO_2_NTs display a distinct tubular structure with a uniform cylindrical morphology and external diameters averaging around 200 nm. While the SEM image suggests these structures, it does not definitively show open-ended lumens due to the resolution and angle of the image. The tubular form typically resulting from the hydrothermal treatment used during their preparation is expected to enhance the membrane’s mechanical strength, selectivity, and permeability by promoting a more homogeneous distribution and alignment within the PES matrix. Furthermore, the aspect ratio and surface roughness of these nanotubes are likely to influence the flux and fouling-resistance of the resultant membranes significantly.

The BET nitrogen adsorption–desorption isotherm analysis is plotted between the partial pressure (p/p_o_) on the x-axis and the volume of sample adsorbed at standard temperature and pressure (STP) on the y-axis; as illustrated in Figure 1g,h, it indicates a distinct type-III isotherm curve for both forms of TiO_2_, signifying their classification as mesoporous materials. For the TiO_2_NTs, BET measurements revealed a specific surface area of 16.187 m^2^/g. Further analyses employing the t-plot method yielded a mean pore diameter of ±0.46 nm and a pore volume of 7.6 × 10^−3^ cm^3^/g. These data points underscore the high surface area and narrower pore size distribution of the NTs, attributes that are conducive to applications requiring high selectivity and permeability. In contrast, the TiO_2_NPs exhibited a lower specific surface area, of 13.322 m^2^/g. The t-plot method indicated a mean pore diameter of ±0.63 nm and a pore volume of 6.9 × 10^−3^ cm^3^/g. Although they also belong to the mesoporous category, the NP have a larger mean pore diameter and a slightly lower surface area compared to the NTs.

The BET results offer a stark contrast between the two TiO_2_ forms, especially in terms of surface area and pore characteristics. The higher surface area and smaller pore diameter of the TiO_2_NTs suggest potential advantages over NPs, particularly in applications for which greater surface area and smaller pore size are beneficial. The BET analyses conclusively delineate the distinct surface and porosity characteristics of the nanofillers. These distinct properties could have significant implications for their respective efficacies in various applications, most notably in membrane technologies. 

### 3.2. Characterization of MMMs

#### 3.2.1. FTIR Analysis

**Figure 2:**
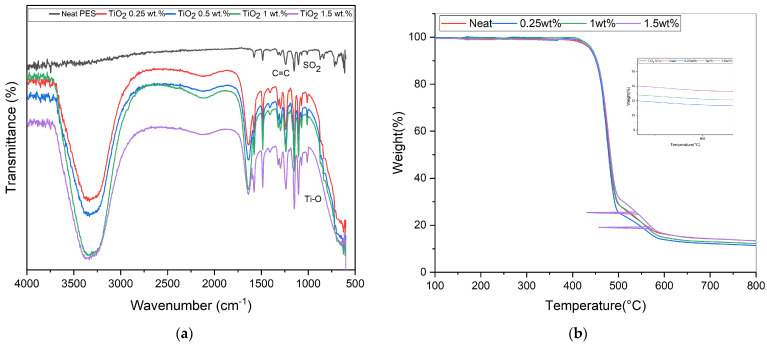
(**a**) FTIR spectra of MMMs with various loadings of TiO_2_NTs; (**b**) TGA analysis of neat PES and MMMs.

The FTIR analysis offers an insightful molecular-level view, revealing the interactions and structural changes occurring within both the neat PES membrane and its TiO_2_NT-modified counterparts. Referring to the spectra presented in Figure 2a, a series of critical peaks stand out, reflecting the chemistry of the membrane materials and their interplay. In the neat PES membrane spectrum, the appearance of a peak at approximately 1486 cm^−1^ is attributed to the C=C stretch, which is a characteristic feature originating from the benzene ring inherent to the PES structure. This peak not only confirms the identity of the PES but also serves as a benchmark when comparing with the modified membranes. Complementing this, the SO_2_ stretching mode, another hallmark of the PES backbone, is observed at around 1241 cm^−1^, further confirming the purity and structural integrity of the unmodified membrane.

Shifting focus to the modified membranes, which incorporate different TiO_2_NT loadings, distinct peaks emerge that signify the successful incorporation of the NTs. A broad band, spanning from 3400 to 3100 cm^−1^, indicates the stretching of the O-H group. This is a direct result of the strong alkaline hydrothermal process utilized during the TiO_2_NT synthesis. The presence of this hydroxyl peak underscores the surface modifications on the NTs which enhance their interaction with the PES matrix. Further down the spectrum, a broad peak, situated in the 500–900 cm^−1^ range, is identified, representing the Ti-O vibrational mode typical of TiO_2_ structures. Its presence in the spectra confirms the integration of TiO_2_NTs into the PES polymer network. Furthermore, a notable observation from the spectra is the amplification of the Ti-O peak’s intensity with increasing TiO_2_NT loadings. This enhancement not only suggests the presence of TiO_2_NTs in the membrane, but also provides a semi-quantitative perspective, hinting at a proportional increase in NT concentration as the loading is elevated.

The TGA results for the neat PES membrane and the MMMs with varying TiO_2_NT loadings are depicted in Figure 2b Analyzing the TGA curves provides valuable insight into the thermal degradation characteristics of these membranes, especially when they are subjected to escalating temperature regimes. While the TGA curves for membranes with different TiO_2_ loadings exhibit qualitative similarities in their thermal degradation behavior, indicating a consistent degradation mechanism, quantitative differences in terms of onset temperatures and rates of weight loss were observed. These differences present the impact of TiO_2_ on the enhancement of the thermal stability of the membranes, although they do not modify the fundamental degradation pathways. This observation suggests that the primary contribution of TiO_2_ integration lies in its effects on properties such as hydrophilicity and mechanical reinforcement, rather than significant alterations to the thermal degradation behavior of the polymer matrix. Up to around 500 °C, the thermal degradation behavior of all three membranes—the neat PES, the 1% TiO_2_NT-loaded MMM, and the 1.5% TiO_2_NT-loaded MMM—remains remarkably similar, which underscores the intrinsic thermal stability of the PES backbone structure across all variants. Beyond this temperature, nuances in the thermal degradation profiles become evident, reflecting the influence of TiO_2_NT loadings. The membrane with 1% TiO_2_NT loading shows superior thermal resilience compared to the neat PES membrane, suggesting that TiO_2_NTs not only enhance hydrophilicity and separation performance but also augment thermal robustness by providing an enhanced thermal barrier, likely due to the inherent stability of the TiO_2_NT structure. Interestingly, while the membrane with a 1.5% TiO_2_NT loading demonstrates better stability than both the neat and the 1% loaded membranes, the improvement is not disproportionately higher. This observation suggests that an optimal balance in thermal shielding effect may be achieved at 1% loading, with NTs uniformly dispersed. The residual mass at high temperatures correlates with the TiO_2_ loadings, indicating that higher concentrations of TiO_2_NTs contribute to greater residual mass, thus providing empirical evidence of their stabilizing effect. An anomalous data point at around 530 °C in the 1.5% TiO_2_NT-loaded membrane reflects an instrumental fluctuation where the temperature momentarily dropped to about 430 °C before returning to 530 °C. This resulted in a horizontal line on the graph which does not affect the overall degradation pattern which resumes from 530 °C, showing a continuation of the established trend.

#### 3.2.2. Membrane Porosity

**Figure 3:**
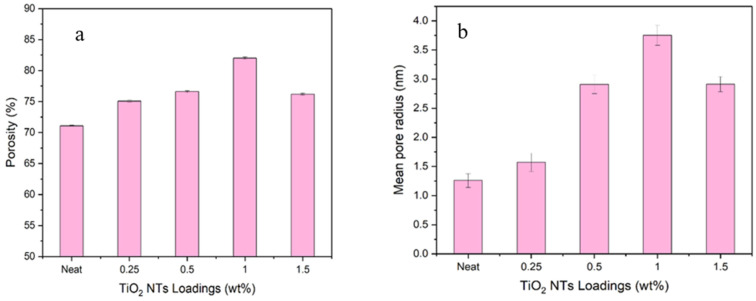
(**a**) Overall porosity; (**b**) mean pore radius of TiO_2_ NTs.

To assess the effects of the incorporation of TiO_2_NT on membrane properties, we investigated the porosity and mean pore radius of both the neat PES and the MMMs, as presented in Figure 3. The data reveal that the porosity of the membranes spans a range of 71–82%, and the mean pore radius varies between 1.25 and 3.75 nm. These parameters are highly dependent on the concentration of TiO_2_NT used as a nanofiller. When the loading of TiO_2_NT was increased, an increase in membrane porosity was observed. The formation of more porous channels in the membrane matrix is attributed to instantaneous de-mixing during the phase inversion process. Furthermore, as the membrane becomes more hydrophilic, larger pores are formed, contributing to increased porosity. Interestingly, a decrease in both porosity and mean pore radius is noted when the NT concentration in the casting solution exceeds 1.5 wt%. This phenomenon can possibly be attributed to the saturation of TiO_2_NT within the membrane matrix, leading to aggregation and the resultant increase in the viscosity of the dope solution.

The incorporation of TiO_2_NT thus significantly alters the porosity and mean pore radius of the membranes, which has important implications for their performance in filtration applications. The elevated porosity at moderate NT concentrations suggests enhanced permeability, which is beneficial for rapid filtration processes. Conversely, the decline in porosity at higher NT concentrations warns of potential limitations, likely due to NT aggregation and increased dope viscosity.

#### 3.2.3. Water Contact Angle

**Figure 4:**
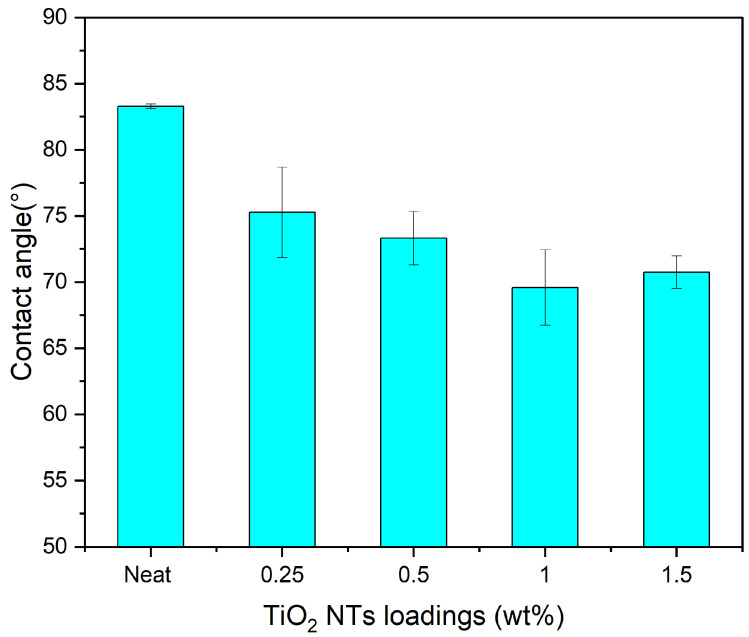
Water contact angle measurements of MMMs.

Understanding the hydrophilic or hydrophobic characteristics of a membrane is crucial for assessing its overall performance. Water contact angle (WCA) measurements offer a reliable metric for such an assessment. As demonstrated in Figure 4, the neat PES membrane had a WCA of 83.2°, which is generally considered to be moderately hydrophilic. The incorporation of TiO_2_NTs led to a noteworthy change in hydrophilicity. Increasing the concentration of TiO_2_NTs from 0.25 to 0.5 wt% resulted in a decrease in the WCA from 75.5° to 73.3°. At concentrations of 1 and 1.5 wt%, the WCAs were further reduced to 69.5° and 70.7°, respectively. This suggests an increasingly hydrophilic membrane surface upon the incorporation of TiO_2_NTs.

The enhanced hydrophilic characteristics can be correlated with the FTIR analysis, where a pronounced stretching of O-H groups was observed for TiO_2_NTs, especially after hydrothermal treatment in alkaline solution. This is indicative of increases in the hydroxyl groups on the surfaces of the TiO_2_NTs. TiO_2_ is inherently hydrophilic, and the alkaline treatment further augments this property by introducing more hydroxyl groups. This synergistic effect substantially lowers the membrane’s interfacial energy, thereby reducing the WCA and making the membrane more hydrophilic. These findings imply that the TiO_2_NTs contribute to both the enhanced hydrophilic properties and the functional group characteristics of the modified PES membranes. The elevated hydrophilicity is generally favorable for increased PWF, higher resistance to protein adsorption, and reduced fouling potential.

#### 3.2.4. Morphological Analysis of Membranes

**Figure 5:**
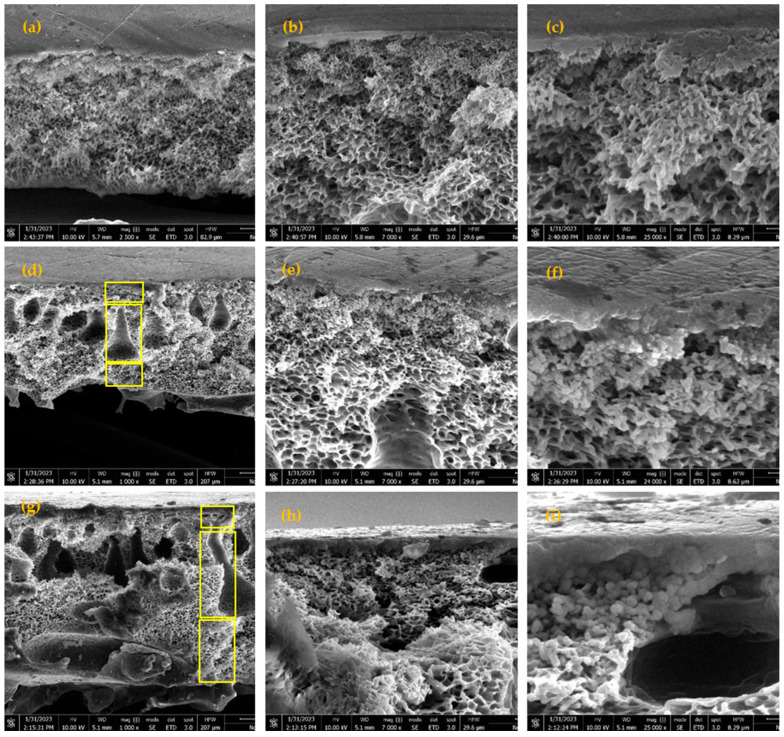
Cross-sectional SEM images of (**a**–**c**) neat PES membrane; (**d**–**f**) 1% TiO_2_NT loading; and (**g**–**i**) 1.5% TiO_2_NT loading.

The cross-sectional morphological examination of the neat PES membranes, alongside those of the MMMs with 1% and 1.5% weight percent (wt%) TiO_2_NT loadings, is depicted in Figure 5 through use of varying magnification levels. This detailed imagery reveals the distinct internal structures that characterize each membrane type, providing essential insights into the impacts of TiO_2_NT integration on membrane architecture and performance.

In all instances, the membranes exhibit a markedly porous structure, underpinned by a thin yet densely constructed top layer. The emergence of this layer can largely be ascribed to the influence of volatile co-solvents employed during the fabrication process, which play a pivotal role in the formation of the membrane’s skin. The pristine PES membrane is characterized by a homogeneously spongy matrix across its entire thickness. This particular morphology is a direct consequence of the phase inversion process’s kinetics—specifically, the delayed de-mixing phenomena attributable to the slow diffusion rates of the non-solvent in regions heavily concentrated with polymer. This spongy structure is crucial, as it underpins the base filtration characteristics of the neat PES membranes, including their permeability and rejection rates.

Upon the introduction of TiO_2_NTs to create MMMs, notable shifts in structural morphology emerge. At loadings of 1 and 1.5 wt%, the membranes exhibit a distinctly asymmetrical architecture comprising the three definitive layers clearly marked in Figure 5d,g:

(i) The uppermost layer is a dense, selective skin which functions as the primary barrier against solute permeation. This layer’s integrity is crucial for defining the membrane’s selectivity.
(ii) Directly beneath this selective layer is a region exhibiting a finger-like structure. This morphology, typically resulting from faster solvent and non-solvent exchange rates, contributes significantly to the overall porosity and permeability of the membrane.
(iii) The bottommost layer, forming the backbone of the membrane, presents a thicker, porous substructure interspersed with macrovoids, which serve to support the membrane’s mechanical strength while also influencing its flow characteristics.

The morphological evolution introduced by the TiO_2_NTs is largely a function of their influence on the kinetics and thermodynamics of the phase inversion process. Specifically, the nanotubes act as pore-forming agents, enhancing the rate of water diffusion within the developing membrane. This acceleration is primarily due to the inherent hydrophilicity of the TiO_2_NTs, which fosters a more rapid exchange between the solvent and non-solvent phases during coagulation. Consequently, with a 1 wt% loading of TiO_2_NTs, there is a noticeable augmentation in the sublayer’s thickness, alongside an elongation and narrowing of the finger-like pores. This structural change suggests that the hydrophilic nature of the TiO_2_NTs not only accelerates the formation of these unique pores but also enhances their interconnectivity, thereby facilitating increased water flux through the membrane. Further, when the concentration of TiO_2_NTs is increased to 1.5 wt%, the structural dynamics shift significantly. The increase in hydrophilicity, coupled with the enhanced viscosity of the casting solution due to the nanotubes, leads to a thicker bottom layer endowed with sponge-like pores. However, at this higher loading, a notable aggregation of TiO_2_NTs, particularly within the top layer, becomes evident. While the hydrophilic properties of the TiO_2_NTs are beneficial up to a point, their aggregation can hinder the efficient transport of water molecules, potentially compromising the membrane’s filtration efficiency. This aggregation may also impact the membrane’s ability to reject solute molecules, as the agglomerated nanotubes could create non-selective pathways through the dense top layer. In light of these findings, it is clear that the incorporation of TiO_2_NTs into the PES matrix profoundly influences the resultant MMMs’ structural and functional attributes. The alterations in membrane morphology induced by various TiO_2_NT loadings highlight the delicate balance between improving hydrophilicity and maintaining an optimal distribution of nanotubes within the polymer matrix to achieve enhanced filtration performance without compromising structural integrity or selectivity.

### 3.3. Membranes’ Performance

#### 3.3.1. Pure Water Permeability

**Figure 6:**
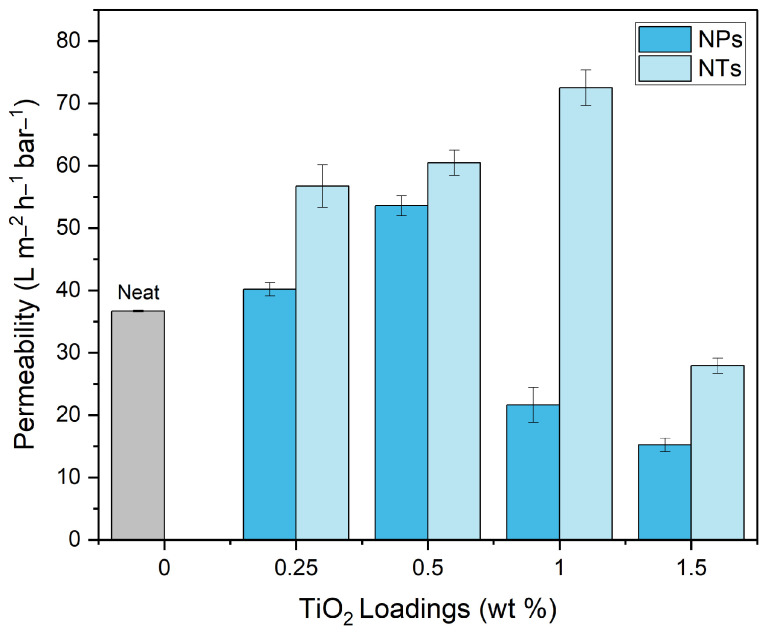
Pure water permeability (PWP) results with TiO_2_ loading.

The assessment of pure water permeability (PWP) serves as a vital indicator for the hydraulic performance of MMMs containing TiO_2_ NPs and NTs. Figure 6 presents PWP values for varying concentrations of TiO_2_ NPs and NTs. For membranes incorporating TiO_2_ NPs, PWP values ranged from 36 to 53 L m^2^ h^−1^ bar^−1^ for loadings from neat to 0.5 wt%. The initial increase in PWP can be attributed to several factors. First, the improved hydrophilicity facilitated by moderate concentrations of TiO_2_ NPs enhances water interaction, thereby boosting the water flux. In addition, the integration of TiO_2_ NPs alters the structural properties of the membrane. These structural changes, including variations in pore size and distribution, contribute significantly to the observed increase in permeability. However, at loadings exceeding 0.5 wt%, both hydrophilicity and structural benefits are overshadowed by adverse effects. NP aggregation at higher concentrations leads to a denser packing within the membrane layers, potentially blocking pore pathways and reducing the effective pore size. This aggregation, combined with increased pore fouling, results in diminished permeability.

Contrastingly, membranes featuring TiO_2_NTs exhibited a more sustained performance improvement, displaying a continual rise in PWP, up to a 1 wt% NT loading. This resulted in a high PWP value of 72 L m^2^ h^−1^ bar^−1^. This consistent improvement can be attributed to the unique, tubular geometry of the TiO_2_NTs, which provides unobstructed and more-efficient water transport pathways. This structure, coupled with the inherent hydrophilicity of the TiO_2_NTs—elucidated further by WCA measurements—favors higher water permeation. However, similar to the TiO_2_NP systems, PWP exhibited a decline at a 1.5 wt% NT loading, reaching 27 L m^2^ h^−1^ bar^−1^. The enhanced viscosity of the casting solution at higher NT concentrations likely disrupts the phase inversion process, consequently affecting the membrane’s microstructure, including porosity and mean pore radius.

In summation, both TiO_2_ NPs and NTs hold potential for improving membrane performance. However, the concentration of these nanostructures should be carefully managed to avoid undesirable outcomes such as pore blockage and reduced flux. While NPs offer initial improvements in hydrophilicity and flux, NTs present a more robust and sustained avenue for performance enhancement, primarily due to their unique morphology and heightened hydrophilicity. By optimizing the concentrations of these TiO_2_ nanostructures, we can strike a balance to achieve superior water treatment capabilities without compromising membrane integrity.

#### 3.3.2. Salts Rejection

**Figure 7:**
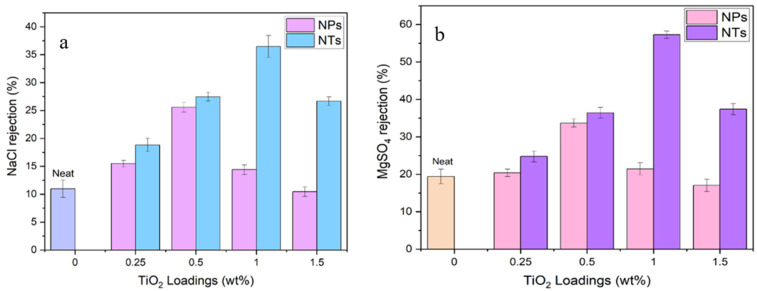
(**a**) NaCl rejection; (**b**) MgSO_4_ rejection.

Salt rejection efficacy was determined in order to evaluate the separation capabilities of the fabricated nanocomposite membranes incorporating TiO_2_NT and TiO_2_NP, as well as those of the neat PES membrane. The data, presented in Figure 7, show a marked improvement in salt rejection for membranes modified with TiO_2_NT as compared to those in which TiO_2_NP is embedded, as well as the neat membrane. In evaluating the superior salt rejection observed in our membranes, the pore size distribution and the structural integrity of the pores are recognized as critical factors. The reduction in large pore defects and a tighter control over pore size distribution contribute significantly to enhancing salt rejection capabilities. These structural features ensure a more selective barrier against salt ions, effectively improving the performance of the membrane. A particular point of interest is the membrane modified with 1 wt% TiO_2_NT, which showed the highest salt rejection rates for both NaCl and MgSO_4_ salts. Specifically, NaCl rejection increased by up to 36%, while MgSO_4_ rejection increased to 59%. It is noteworthy that all NF membranes exhibited higher rejection for MgSO_4_ as compared to NaCl. This is predominantly due to the fact that divalent ions like Mg^2+^ and SO_4_^2−^ are more readily rejected than monovalent ions such as Na^+^ and Cl^−^. This ion-specific rejection is attributable to the greater ionic radius and charge of divalent ions, consistent with the Donnan effect, which influences the surface charge distribution on the MMMs. This observation is similar to those in previous studies that have reported similar findings [41,42].

The higher salt rejection performance with TiO_2_NT correlates with the improved membrane hydrophilicity, effective pore sizes, and porosity that we observed in the preceding sections. The superior hydrophilic nature of TiO_2_NT, validated by WCA measurements and FTIR data, appears to play a key role in achieving higher salt rejection. In summary, the salt rejection performance reveals that TiO_2_NT-modified membranes provide efficient ion separation, but optimal performance is achieved at a specific loading concentration. It emphasizes the need for a balanced TiO_2_NT concentration to achieve maximal salt rejection without compromising other membrane properties.

#### 3.3.3. Molecular Weight Cut-Off and Dye Rejection Performance of Modified Membranes

Water-soluble dyes in industrial wastewater are environmental pollutants that necessitate effective treatment methods. In this context, NF MMMs fabricated with various loading of TiO_2_NT, owing to their superior salt rejection and water permeability, were tested for their ability to remove different dyes. Three dyes of varying molecular weights were selected: Methylene Blue (MB: 373 g/mol), Congo Red (CR: 696 g/mol), and Rose Bengal (ROB: 1017 g/mol). The chosen dyes were expected to offer insights into the membrane’s rejection capabilities across a molecular weight range, thereby also allowing the assessment of the membrane’s MWCO.

**Figure 8:**
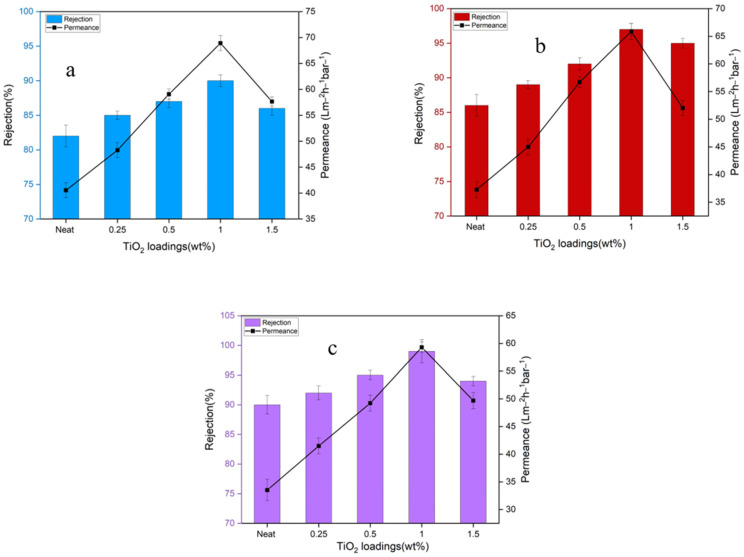
(**a**) MB rejection; (**b**) CR rejection; and (**c**) RB rejection.

Dye rejection performance is illustrated in Figure 8. The membrane loaded with 1 wt% TiO_2_NT showed remarkable efficiency, rejecting 82–90% of MB, 86–97% of CR, and 90–99% of ROB. Notably, dye rejection was higher for dyes with greater molecular weight, underlining the role of molecular size in membrane performance. The incorporation of TiO_2_NT effectively blocked dye molecules’ passage, elevating solute rejection levels. The type of interaction between the membrane surface and the dyes—specifically, attraction for positively charged MB and repulsion for negatively charged CR and ROB—also played a role in dye rejection.

**Figure 9:**
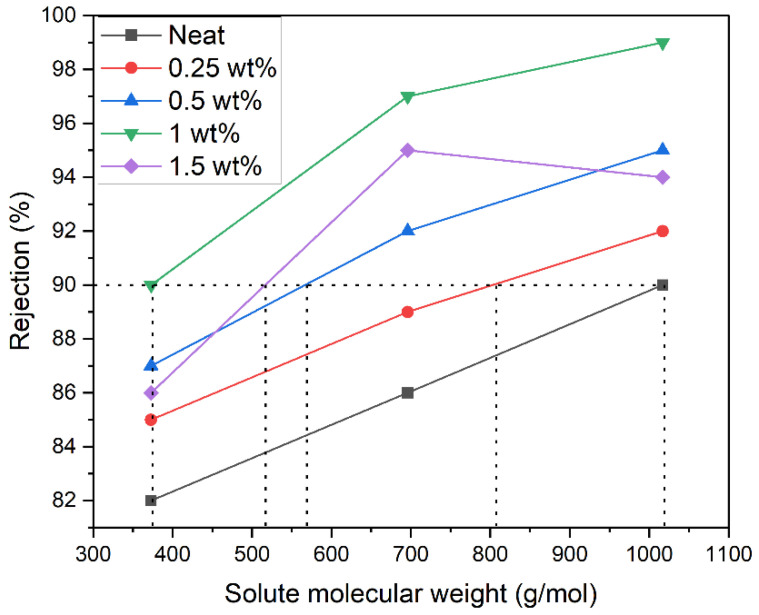
MWCOs of MMMs.

To assess the membranes for industrial applications, the MWCO was determined; it is presented in Figure 9. Using dyes of different charge types and molecular weights, the MWCO was found to decrease with increasing TiO_2_NT loading: 802 g/mol at 0.25 wt%, 572 g/mol at 0.5 wt%, and 373 g/mol at 1 wt%. The optimized membrane with 1 wt% TiO_2_NT loading is therefore best suited for rejecting pollutants with a molecular weight greater than 373 g/mol. The MWCO and dye rejection studies further attest to the efficacy of TiO_2_NT in enhancing membrane performance. The improvement in MWCO specifically relates back to the increased hydrophilicity and optimized pore sizes and validates the utility of TiO_2_NT-modified membranes in advanced filtration applications, including dye removal. 

#### 3.3.4. Long-Term Stability Assessment of Optimized TiO_2_NT/PES MMMs

**Figure 10:**
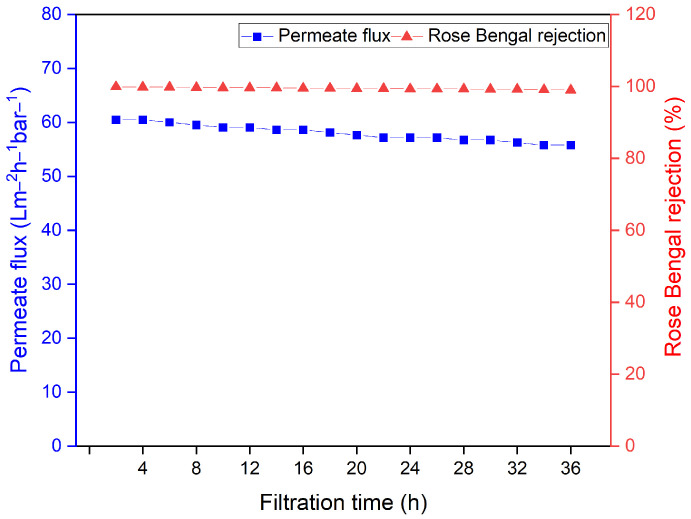
Long-term assessment of membrane performance.

The membrane incorporating 1 wt% TiO_2_NT was selected for long-term stability testing due to its superior performance in dye rejection and water permeability. The membrane’s long-term operational stability was assessed using a 36 h continuous filtration test, focusing on Rose Bengal dye permeation and retention flux. Figure 10 shows the time-dependent changes in these parameters. Initial permeate flux was observed to be 60 L/m^2^ h, which gradually declined to 55 L/m^2^ h over the 36 h test period. Notably, despite this decrease, the dye removal efficiency remained consistently high, at around 99%. The observed decline in permeate flux is attributed to an increase in concentration polarization, which creates resistance to membrane filtration due to the aggregation of dye molecules on the feed side. However, the flux only reduced by approximately 7.5% from the 3rd to the 36th h of the filtration test. This modest decline indicates the effectiveness of TiO_2_NT in resisting membrane fouling, confirming its significant role in enhancing long-term operational stability. 

#### 3.3.5. Membrane Anti-Fouling Analysis

**Figure 11:**
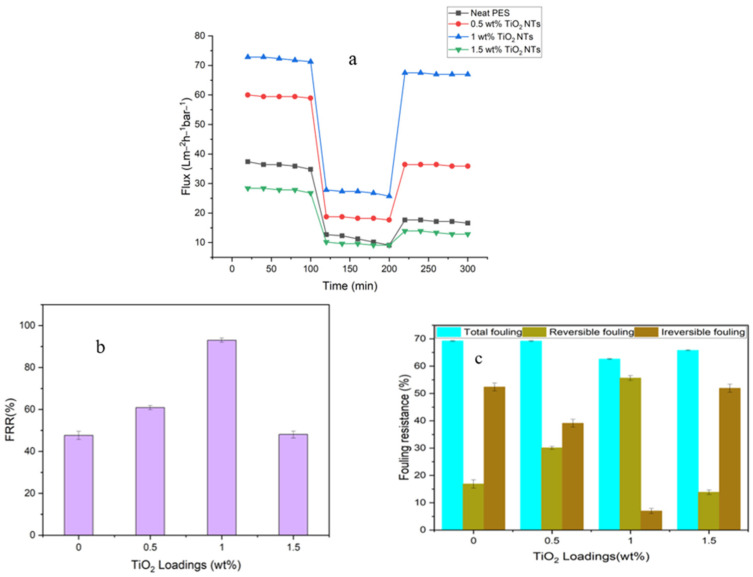
(**a**) Flux versus time for TiO_2_ NT-blended PES membranes at a pressure of 8 bar during three steps: (i) pure water flux for 120 min before washing; (ii) 1000 ppm BSA solution flux for 80 min; and (iii) pure water flux for 100 min after 30 min washing with distilled water. (**b**) Flux recovery ratio (FRR) and (**c**) fouling resistance ratio for prepared membranes.

Fouling remains a critical bottleneck in membrane technology, reducing membrane lifespan, elevating maintenance costs, and impeding performance [43]. Figure 11a–c displays the BSA solution (1000 ppm) anti-fouling tests for membranes with different TiO_2_NT loadings, revealing a set of remarkable quantitative metrics that validate their enhanced anti-fouling capabilities. In the presentation of the fouling results in Figure 11, variability is observed between different membrane samples. This variability can be attributed to variations in the dispersion of TiO_2_ nanotubes within the polymer matrix, which result in differences in pore size and pore size distributions. These factors can influence the fouling behavior of each membrane, affecting the consistency of the results. 

In terms of FRR, the membrane with 1 wt% TiO_2_NT loading showed the highest FRR value, at 93%, a significant increase compared to the neat PES membrane’s 47%. This indicates a robust ability to recover the original water flux after fouling events, implying enhanced operational longevity. The total fouling (R_t_) was lowered upon TiO_2_NT incorporation. While neat and 1.5 wt%-loaded membranes exhibited R_t_ values of 69 and 65%, respectively, it was reduced to 62% in the case of the 1 wt% TiO_2_NT-loaded membrane, further underscoring the anti-fouling effectiveness of TiO_2_NTs. In conjunction with this, the reversible fouling (R_r_) increased from 16% for the neat PES, and 13% for the 1.5 wt% loaded membrane, to 55% for the 1 wt% TiO_2_NT-loaded membrane. This indicates that fouling is predominantly reversible and can be managed with routine cleaning procedures, reducing downtime and operational costs. Most notably, the irreversible fouling (R_ir_) was reduced dramatically, dropping from 52% in neat and 51% in 1.5 wt% loaded membranes to a low of 6.9% for the 1 wt% TiO_2_NT-loaded membrane. This low R_ir_ implies that the membrane is highly efficient in minimizing the most problematic form of fouling—irreversible fouling. 

One of the underlying theories behind the anti-fouling mechanism of TiO_2_NTs considers their hydrophilicity. When integrated into a polymer matrix, TiO_2_NTs significantly increase the membrane’s surface hydrophilicity. It is well-established that hydrophilic membranes discourage protein adsorption, consequently preventing biofouling at the molecular level. The surface morphology and pore structure of TiO_2_NT-embedded membranes also contribute to anti-fouling. TiO_2_NTs provide a more porous, open structure which facilitates rapid water transport while simultaneously limiting the passage of foulants. This pore morphology offers an additional physical barrier to fouling, thereby enhancing the membrane’s operational efficiency. The electrostatic interactions between the membrane surface and foulants also deserve consideration. TiO_2_NTs can create a net negative charge on the membrane surface, thereby repelling similarly charged foulants and thus further enhancing their anti-fouling properties. These findings have significant long-term implications. Reduced fouling will extend membrane life, decrease the frequency of cleaning, reduce energy demands, and, overall, make the membrane technology more sustainable and economically viable. 

## 4. Conclusions

This study successfully synthesized TiO_2_NTs through an alkaline hydrothermal treatment process, subsequently embedding them, along with TiO_2_NPs, into PES to prepare nanocomposite mixed matrix membrane (MMM) via the phase inversion method. A multi-dimensional evaluation encompassing pure water flux, salt rejection, dye removal, and fouling parameters was conducted to gauge the performance of these modified membranes relative to unmodified (neat) PES membranes. Owing to their unique tubular structure, TiO_2_NTs notably enhanced the membrane’s hydrophilicity and surface properties. This contributed to marked improvements in both permeability and selectivity, with an increase in flux from 36 to 72 L m^−2^ h^−1^ bar^−1^ at 1 wt% loading of TiO_2_NTs in the MMMs. Impressively, the Rose Bengal rejection rate remained consistently high, at around 99%. The optimized membrane, comprising 1 wt% TiO_2_NT loading, was further subjected to long-term stability testing. Over a 36 h stability assessment, the membrane exhibited only a minor decrement in permeate flux while maintaining a constant dye removal efficiency. Anti-fouling tests revealed minimal fouling, with irreversible fouling recorded at a mere 6.9%, indicating a high level of anti-fouling performance. MMM with a 1 wt% TiO_2_NT-loaded membrane emerged as the most promising candidate, with superior filtration capabilities, long-term operational stability, and robust anti-fouling characteristics. This study paves the way for the future development of high-performance filtration membranes, laying a strong foundation for subsequent research and potential industrial applications.

## Figures and Tables

**Table 1:** Composition of membranes (note, all samples contain PES at 20 wt%, DMF at 60 wt%, and 1,4-dioxane at 20 wt%).

**Membrane Type**	**TiO_2_NPs, NTs (wt%)**
Neat PES	0
TiO_2_ (0.25)	0.25
TiO_2_ (0.5)	0.5
TiO_2_ (1)	1
TiO_2_ (1.5)	1.5

## Data Availability

The raw data supporting the conclusions of this article will be made available by the authors on request.

## Supplementary Materials

The following supporting information can be downloaded at: https://www.mdpi.com/2077-0375/14/5/116/s1, Figure S1: Schematic illustration of synthesis process of TiO_2_ NTs; Figure S2: Schematic illustration of fabrication of TiO_2_ NTs/NPs based MMMs.
