# Supplementary material for: Enhancing Water Purification by Integrating Titanium Dioxide Nanotubes into Polyethersulfone Membranes for Improved Hydrophilicity and Anti-Fouling Performance
Source: Membranes (Basel). 2024 May 17;14(5):116. doi: 10.3390/membranes14050116 (PMC11123263; doi:10.3390/membranes14050116)
Supplement: Supplementary file 1 [file membranes-14-00116-s001.zip › membranes-2944914-supplementary.pdf]

membranes

Supplementary Materials

Enhancing Water Purification by Integrating Titanium Dioxide Nanotubes into Polyethersulfone Membranes for Improved Hydrophilicity and Anti-Fouling Performance

Ayesha Bilal [1], Muhammad Yasin [2,*], Faheem Hassan Akhtar [3], Mazhar Amjad Gilani [1], Hamad Alhmohamadi [4], Mohammad Younas [5], Azeem Mushtaq [2,6], Muhammad Aslam [2], Mehdi Hassan [7], Rab Nawaz [8], Aqsha Aqsha [9], Jaka Sunarso [10], Muhammad Roil Bilad [11] and Asim Laeeq Khan [4,*]

1 Department of Chemistry, COMSATS University Islamabad, Lahore Campus, Lahore 54000, Pakistan;
2 Department of Chemical Engineering, COMSATS University Islamabad, Lahore Campus, Lahore 54000, Pakistan
3 Department of Chemistry and Chemical Engineering, Lahore University of Management Sciences (LUMS), Lahore 54792, Pakistan
4 Department of Chemical Engineering, Faculty of Engineering, Islamic University of Madinah, Madinah 42351, Saudi Arabia
5 Department of Chemical Engineering, Faculty of Mechanical, Chemical and Industrial Engineering, University of Engineering and Technology, Peshawar 25120, Pakistan
6 School of Energy and Environment, City University of Hong Kong, Kowloon Tong, Hong Kong SAR, China
7 Department of Chemistry, University of Baltistan, Skardu 16100, Pakistan; mehdi.hassan@uobs.edu.pk
8 Center for Applied Mathematics and Bioinformatics (CAMB), Gulf University for Science and Technology, Hawally 32093, Kuwait
9 Department of Bioenergy Engineering and Chemurgy, Faculty of Industrial Technology, Institute Teknologi Bandung, Bandung 40132, Indonesia
10 Research Centre for Sustainable Technologies, Faculty of Engineering, Computing and Science, Swinburne University of Technology, Jalan Simpang Tiga, Kuching 93350, Sarawak, Malaysia
11 Faculty of Integrated Technologies, Universiti Brunei Darussalam, Gadong BE 1410, Brunei
* Correspondence: myasin@cuilahore.edu.pk (M.Y.); akhan@iu.edu.sa (A.L.K.)

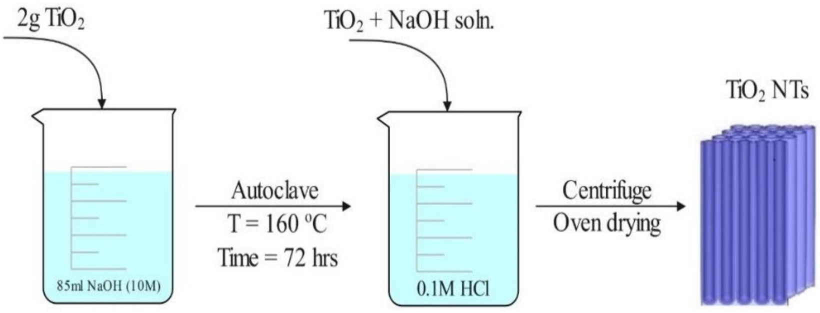

Figure S1. Schematic illustration of synthesis process of TiO_2 NTs.

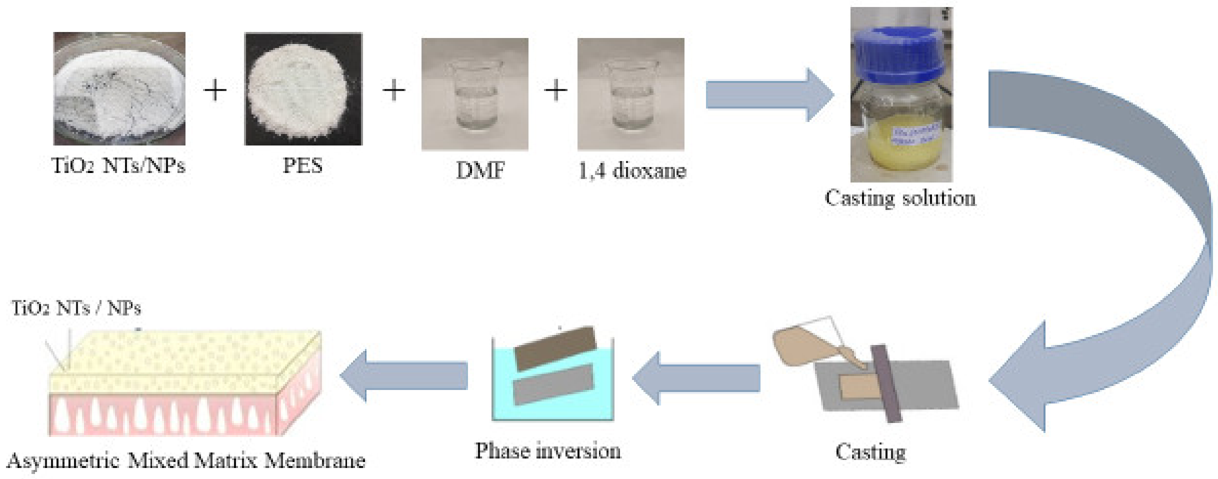

Figure S2. Schematic illustration of fabrication of TiO_2 NTs/NPs based MMMs.
